# Improved prediction of incident vertebral fractures using opportunistic QCT compared to DXA

**DOI:** 10.1007/s00330-019-06018-w

**Published:** 2019-02-21

**Authors:** Maximilian T. Löffler, Alina Jacob, Alexander Valentinitsch, Anna Rienmüller, Claus Zimmer, Yu-Mi Ryang, Thomas Baum, Jan S. Kirschke

**Affiliations:** 1 Department of Neuroradiology, Klinikum rechts der Isar, Technische Universität München, Ismaninger Str. 22, 81675 Munich, Germany; 2 Department of Neurosurgery, Klinikum rechts der Isar, Technische Universität München, Munich, Germany; 3 Department of Orthopedic and Trauma Surgery, Medical University Vienna, Vienna, Austria

**Keywords:** Bone density, Osteoporosis, Spinal fractures, Photon absorptiometry, Multidetector computed tomography

## Abstract

**Objectives:**

To compare opportunistic quantitative CT (QCT) with dual energy X-ray absorptiometry (DXA) in their ability to predict incident vertebral fractures.

**Methods:**

We included 84 patients aged 50 years and older, who had routine CT including the lumbar spine and DXA within a 12-month period (baseline) as well as follow-up imaging after at least 12 months or who sustained an incident vertebral fracture documented earlier. Patients with bone disorders aside from osteoporosis were excluded. Fracture status and trabecular bone mineral density (BMD) were retrospectively evaluated in baseline CT and fracture status was reassessed at follow-up. BMD_QCT_ was assessed by opportunistic QCT with asynchronous calibration of multiple MDCT scanners.

**Results:**

Sixteen patients had incident vertebral fractures showing lower mean BMD_QCT_ than patients without fracture (*p* = 0.001). For the risk of incident vertebral fractures, the hazard ratio increased per SD in BMD_QCT_ (4.07; 95% CI, 1.98–8.38), as well as after adjusting for age, sex, and prevalent fractures (2.54; 95% CI, 1.09–5.90). For DXA, a statistically significant increase in relative hazard per SD decrease in *T*-score was only observed after age and sex adjustment (1.57; 95% CI, 1.04–2.38). The predictability of incident vertebral fractures was good by BMD_QCT_ (AUC = 0.76; 95% CI, 0.64–0.89) and non-significant by *T*-scores. Asynchronously calibrated CT scanners showed good long-term stability (linear drift ranging from − 0.55 to − 2.29 HU per year).

**Conclusions:**

Opportunistic screening of mainly neurosurgical and oncologic patients in CT performed for indications other than densitometry allows for better risk assessment of imminent vertebral fractures than dedicated DXA.

**Key Points:**

*• Opportunistic QCT predicts osteoporotic vertebral fractures better than DXA reference standard in mainly neurosurgical and oncologic patients.*

*• More than every second patient (56%) with an incident vertebral fracture was misdiagnosed not having osteoporosis according to DXA.*

*• Standard ACR QCT-cutoff values for osteoporosis (< 80 mg/cm* ^*3*^ *) and osteopenia (≤ 120 mg/cm* ^*3*^ *) can also be applied scanner independently in calibrated opportunistic QCT.*

## Introduction

Osteoporosis is a metabolic bone disease leading to reduced bone strength and manifesting in low-energy fractures [1]. Resulting pain and disability pose a huge burden on patients and society [2, 3]. Effective prevention and medical treatment for osteoporosis exist [4], but are not initiated in many patients [3, 5], partly because bone densitometry is under-utilized [6, 7]. Bone mineral density (BMD) as the single most important parameter accounts for approximately 70% of bone strength [8].

For the diagnosis of osteoporosis, the up-to-date reference standard in clinical bone densitometry is dual energy X-ray absorptiometry (DXA) [9]. This projectional technique is performed at the spine and hip in order to formulate a diagnosis based on a WHO normative population [10]. However, the role of DXA in the diagnosis of osteoporosis can be put into question, given that in a large population-based study, less than half of women (44%) and even fewer men (21%) of all individuals with prevalent osteoporotic fractures were correctly diagnosed with osteoporosis by DXA [11].

Quantitative CT (QCT) is a notable alternative to DXA with at least the same ability to predict vertebral fractures in women, although it is not officially approved to diagnose osteoporosis, since the WHO classification relies on *T*-scores derived by DXA [12]. QCT is a non-projectional technique performed on clinical CT scanners to measure volumetric BMD. Due to its three-dimensional characteristic, QCT is largely independent of degenerative changes in the spine and can differentiate between cortical and trabecular bone. Trabecular bone is about eight times more metabolically active than cortical bone and therefore prone to changes in osteoporosis [12]. Osteodensitometry in routine CT scans, which have been acquired for other purposes, can distinguish osteoporotic from healthy individuals [13] and bears a huge potential of opportunistic screening [14]. Accordingly, densitometry based on non-dedicated CT scans is named opportunistic QCT. In the following, “BMD” will refer to *volumetric BMD* as assessed by opportunistic QCT—not DXA measured areal BMD—as previously encouraged [12]. Wherever helpful for the reader to avoid confusion, we explicitly identify BMD_QCT_ as being derived from CT measurements.

The comparative potential of QCT and DXA to discriminate between patients with and without prevalent vertebral fractures has been investigated in many cross-sectional studies [15–18]. Recently, the risk of future vertebral fractures has been investigated in opportunistic CT data [19]; however, DXA data was not included. In the present study, we investigate the association between the risk of future osteoporotic vertebral fractures and opportunistic BMD measurements in routine CT scans acquired for other purposes compared to measurements of the reference standard DXA.

## Methods

### Study population

**Fig. 1:**
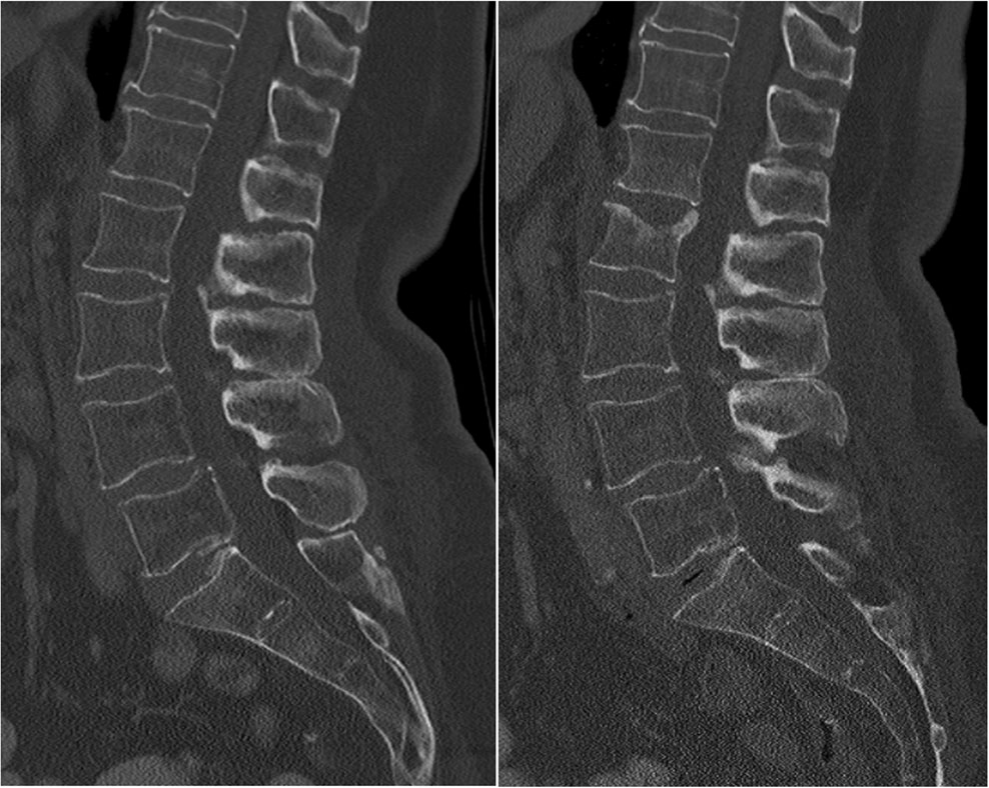
Left: baseline CT of a 72-year-old female patient with osteopenia according to DXA (*T* = − 1.7) and osteoporosis according to opportunistic QCT (BMD = 70.5 mg/cm^3^). Right: in follow-up after 5.2 years, the patient had sustained an incident vertebral compression fracture of L2

The local institutional review board approved this retrospective study and waived written informed consent. In a formal query on the institutional database including all patients registered until May 2017, we identified 325 patients aged 50 years and older at DXA, who had DXA and baseline CT including the lumbar spine within 12 months. After excluding patients for several reasons (history of vertebral metastasis or hematologic disorder [16], CT on a scanner without calibration or with different tube voltage setting [10], and CT without at least one lumbar vertebra assessable for densitometry [6]), 84 patients with follow-up imaging after at least 12 months or who sustained an incident vertebral fracture documented in an earlier imaging study were included. These patients had routine CT for a variety of indication other than densitometry (36% acute back pain or suspected spinal fracture; 30% staging, restaging, or malignoma follow-up; 15% exclusion of acute abdominal pathology; 12% chronic back pain; and 7% postoperative CT after neurosurgery). An incident vertebral fracture was defined as a fracture that occurred either in a previously non-fractured vertebra (Fig. 1) or in an already fractured, consolidated vertebra with increase in at least one grade of the semiquantitative scale by Genant [20]. Consolidation was ensured by the absence of bone marrow edema in recent MR imaging. Active, progressive vertebral fractures (with bone marrow edema in MRI or signs of callus formation in CT [21]) were not considered as incidental fractures as they are usually associated with continuous clinical symptoms [22].

### Dual energy X-ray absorptiometry

DXA measurements were performed on a single densitometer (GE Lunar Prodigy, GE Healthcare) by trained technologists and quality was assured through evaluation by experienced physicians supervised by a certified densitometrist. Total proximal femur of both hips and lumbar vertebrae L1 to L4 was assessed in anterior-posterior projection [23]. Those skeletal sites affected by severe local structural change or artifact were excluded. If only one vertebra remained after exclusion of other vertebrae, the measurement was solely based on the hip. The overall lowest *T*-score at the lumbar spine or total proximal femur was reported and accounted for a single diagnosis of osteoporosis [24]. Osteoporosis was defined as *T* ≤ − 2.5 standard deviations (SD), osteopenia as − 2.5 < T ≤ − 1 SD [9].

### Computed tomography

Baseline CT was performed on five multidetector computed tomography (MDCT) scanners in the same hospital (Philips Brilliance 64 and iCT 256, Philips Medical Care; Siemens Somatom Definition AS+, Definition AS, and Sensation Cardiac 64, Siemens Healthineers), partly with administration of oral (Barilux Scan, Sanochemia Diagnostics) and intravenous contrast medium (Imeron 400, Bracco). Image data was acquired in helical mode with a peak tube voltage of 120 kVp, a slice thickness of 0.9 to 1 mm and adaptive tube load. Sagittal reformations with a slice thickness of 2 mm and standard bone kernel were reconstructed, as proposed for better fracture detection [25].

### Opportunistic QCT

Asynchronous QCT was performed in baseline CT, a technique that provides results comparable to conventional QCT [26]. Attenuation values in HU were manually sampled with tools of the institutional picture archiving and communication system software (Sectra IDS7, Sectra AB) and transformed into volumetric BMD with conversion equations calculated by asynchronous calibration. An experienced radiologist placed a circular region of interest in trabecular bone of lumbar vertebrae L1 to L4, as previously described [27], using on-the-fly calculated midsagittal stacks of 15-mm thickness. Sampled HU was averaged over assessed vertebrae, omitting fractured vertebra or those with apparent alterations of the trabecular bone due to degeneration or hemangioma.

**Fig. 2:**
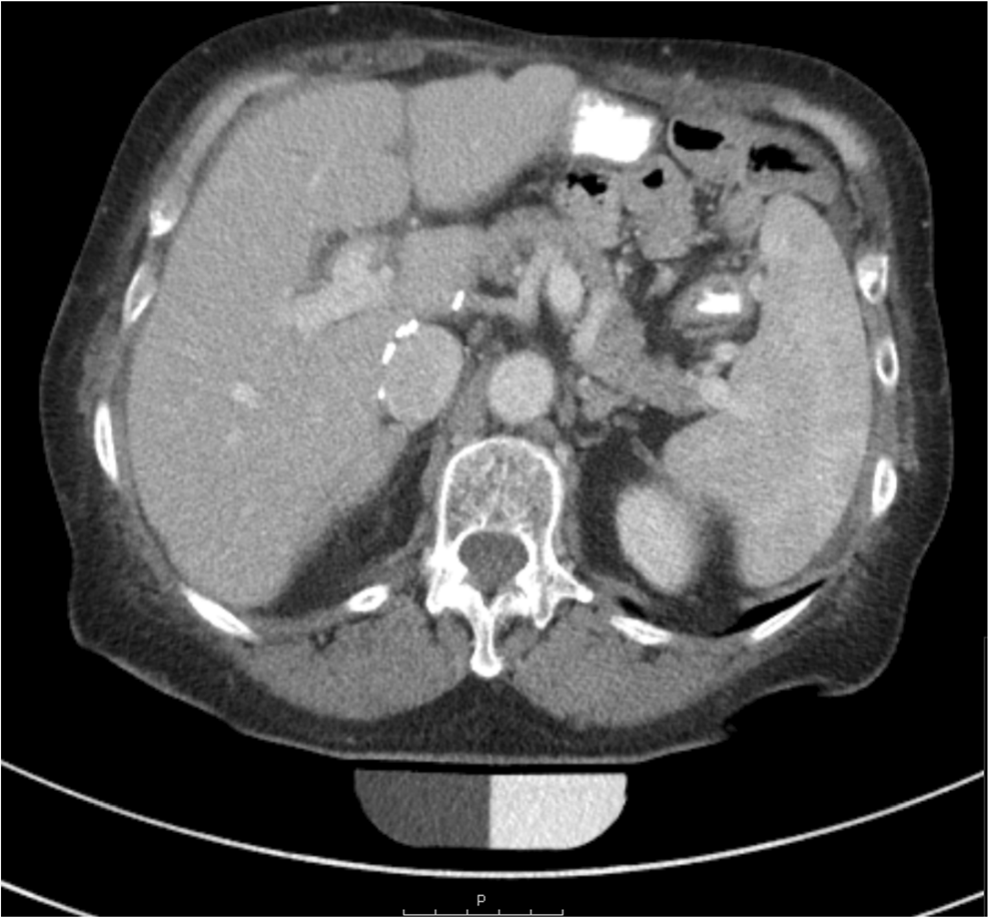
Routine CT scan of a 63-year-old female patient for follow-up purpose after metastatic gastric cancer and liver transplant with administration of oral and intravenous contrast medium in portal venous phase. For two MDCT scanners (Siemens Somatom Definition AS [in this example] and Sensation Cardiac 64), retrospective measurements of an in-plane calibration phantom present underneath patients during routine scans were used for asynchronous calibration and evaluation of long-term scanner stability

HU-to-BMD conversion equations were calculated by linear regression, in three scanners (Philips Brilliance 64, iCT 256, and Siemens Somatom Definition AS+) based on measurements of density-reference phantoms (QRM) in dedicated scans with the same tube voltage and scanner settings as in clinical routine acquisitions, and in two already decommissioned scanners (Siemens Somatom Definition AS and Sensation Cardiac 64) based on retrospective measurements of a density-reference phantom (Osteo Phantom, Siemens Healthineers), which had been included in the scanner couch during clinical CT scans for a certain period of time in the past (Fig. 2). Retrospective measurements of the Siemens Osteo phantom and a second calibration phantom (Mindways Software) were performed in CT exams, which were randomly selected from the institutional database in 2-month intervals over the entire time period when phantoms were present. Thereby, long-term scanner stability was evaluated in three scanners (Philips iCT 256, Siemens Somaton Definition AS, and Sensation Cardiac 64). Conversion equations and long-term stability measures are shown in Table 4. A BMD correction offset for contrast-enhanced CT scans with arterial (− 8.6 mg/cm^3^) and portal venous contrast phase (− 15.8 mg/cm^3^) was added based on previous investigations [28]. Osteoporosis was defined as BMD < 80 mg/cm^3^ and osteopenia as 80 ≤ BMD ≤ 120 mg/cm^3^ [29].

### Statistical analysis

Baseline characteristics were compared using *t* test for continuous variables and chi-square test for categorical variables. In Cox proportional hazard models, hazard ratio (HR) and 95% confidence interval (CI) for the risk of incident vertebral fractures were calculated, firstly with unadjusted BMD_QCT_ and DXA *T*-score, and secondly with age at DXA, sex, and prevalent fractures as covariates. For better comparability, HR is expressed per SD decrease in BMD_QCT_ or DXA *T*-score. In Kaplan-Meier curves, fracture-free time periods were visualized for patients with osteoporosis, osteopenia, or normal bone density defined either by BMD_QCT_ or DXA *T*-score. In ROC analysis, AUC was calculated to predict incident vertebral fractures by BMD_QCT_ and DXA *T*-scores. In order to assess long-term scanner stability, slope of linear regression (SL) and coefficient of variation of the standard error of the estimate (CV) were calculated for measurements of two phases of the calibration phantoms. For each scanner, CV was averaged by the root-mean-square [30]. All statistical analyses were conducted with IBM SPSS Statistics 25 (IBM), with an *α*-level of significance *p* < 0.05.

## Results

Over a median follow-up of 2.6 years (interquartile range 1.7–3.6 years), 16 of 84 patients (19%) sustained an incident vertebral fracture (Table 1). Patients with incident vertebral fracture were significantly older with a mean age of 73.9 ± 7.4 years and had a lower mean BMD_QCT_ of 56.7 ± 31.6 mg/cm^3^ than patients without fracture with a mean age of 67.7 ± 8.6 years (*p* = 0.01) and a mean BMD_QCT_ of 93.3 ± 41.7 mg/cm^3^ (*p* = 0.001). The number of patients defined as osteoporotic by BMD_QCT_ differed significantly between patients with and without incident vertebral fractures (*p* = 0.004). However, there was no significant difference in DXA *T*-score between patients with and without incident vertebral fractures (*p* = 0.179). Seven of 16 patients (44%) with incident vertebral fractures were correctly diagnosed with osteoporosis according to DXA, whereas 13 of 16 (81%) were categorized having osteoporotic BMD_QCT_.

**Table 1:** Baseline characteristics of patients with and without incident vertebral fractures

		No incident vertebral fracture (*n* = 68)	Incident vertebral fracture (*n* = 16)	No vs. incident vertebral fracture	All (*n* = 84)
Women, *n* (%)		54 (79%)	13 (81%)	n.s.	67 (80%)
Age at DXA, mean (SD)		67.7 (8.6)	73.9 (7.4)	*p* = 0.01	68.9 (8.7)
Days between DXA and CT, median (range)		70 (0–362)	34 (0–350)	n.s.	62 (0–362)
Days to follow-up imaging, median (range)		1018 (373–2425)	768 (19–1891)	*p* = 0.049	935 (19–2425)
Non-enhanced CT scans, *n* (%)		33 (49%)	7 (44%)	n.s.	40 (48%)
Diagnosis by lumbar DXA, *n* (%)		39 (57%)	10 (63%)	n.s.	49 (58%)
BMD by QCT, mean (SD)		93.3 (41.7)	56.7 (31.6)	*p* = 0.001	86.3 (42.4)
DXA *T*-score, mean (SD)		− 1.6 (1.7)	− 2.2 (1.8)	n.s.	− 1.7 (1.7)
Maximum Genant grade of prevalent fractures, *n* (%)	No fracture	35 (51%)	4 (25%)	n.s.	39 (46%)
	Grade 1	10 (15%)	1 (6%)	n.s.	11 (13%)
	Grade 2	12 (18%)	4 (25%)	n.s.	16 (19%)
	Grade 3	11 (16%)	7 (44%)	*p* = 0.016	18 (22%)
Bone density by QCT, *n* (%)	Normal	15 (22%)	1 (6%)	n.s.	16 (19%)
	Osteopenia	25 (37%)	2 (13%)	n.s.	27 (32%)
	Osteoporosis	28 (41%)	13 (81%)	*p* = 0.004	41 (49%)
Bone density by DXA, *n* (%)	Normal	22 (32%)	2 (12%)	n.s.	24 (29%)
	Osteopenia	24 (35%)	7 (44%)	n.s.	31 (37%)
	Osteoporosis	22 (33%)	7 (44%)	n.s.	29 (34%)

*SD*, standard deviation; *n.s.*, non-significant at the *α*-level *p* < 0.05

**Fig. 3:**
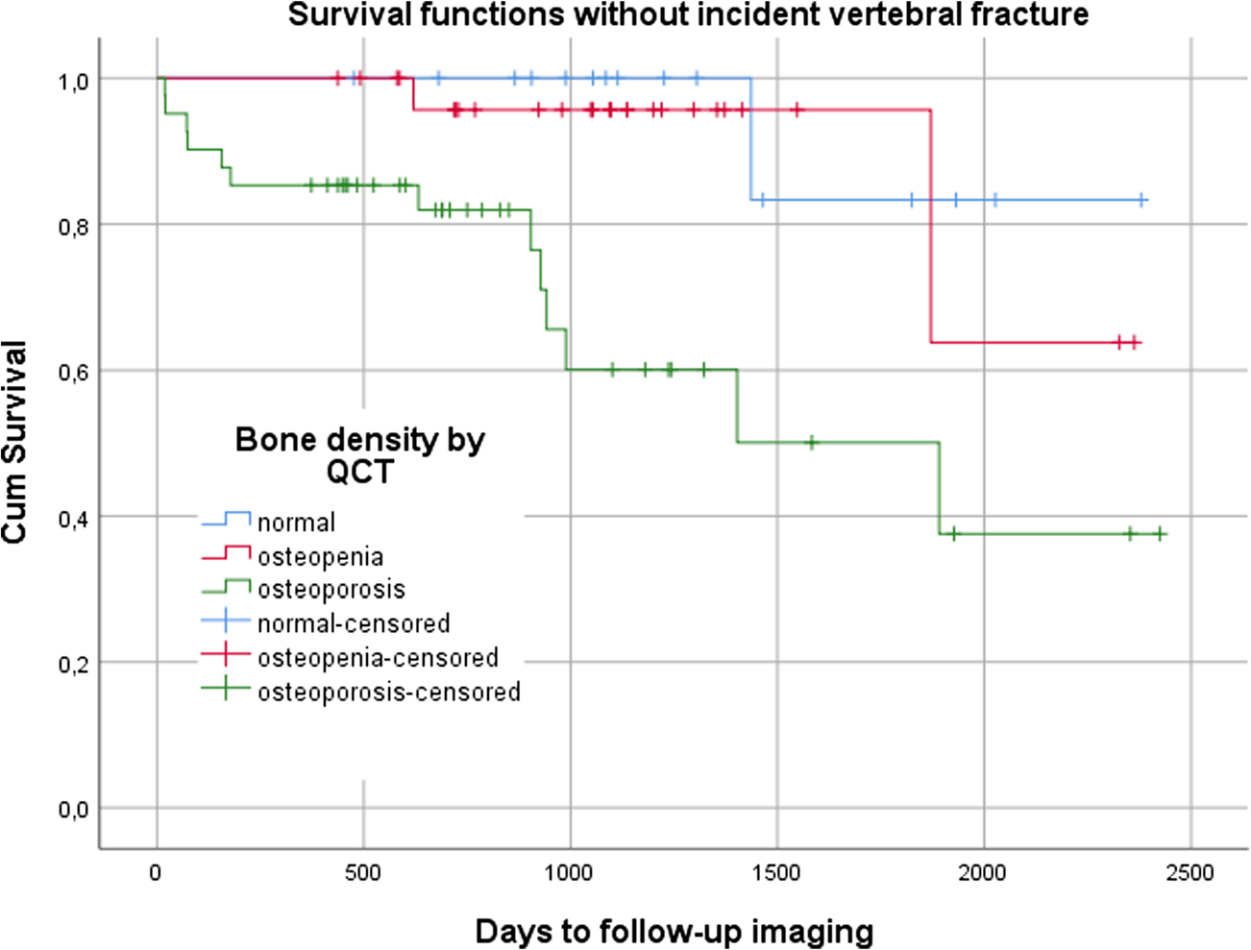
Kaplan-Meier curves of time periods without an incident vertebral fracture stratified by opportunistic QCT into patients with normal (> 120 mg/cm^3^), osteopenic (80 ≤ BMD ≤ 120 mg/cm^3^), or osteoporotic BMD (< 80 mg/cm^3^)

**Fig. 4:**
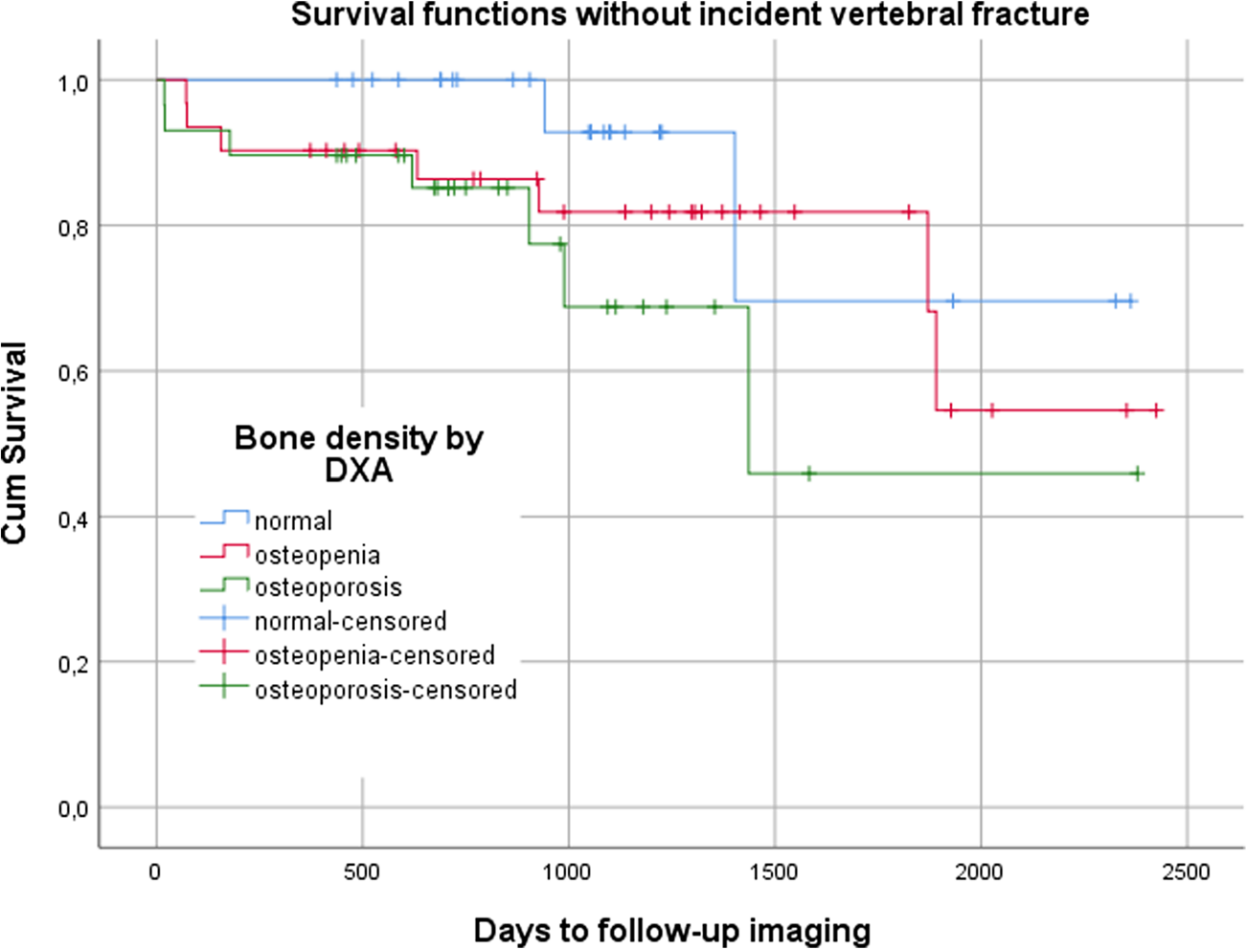
Kaplan-Meier curves of time periods without an incident vertebral fracture stratified by DXA into patients with normal bone mass (T > − 1), osteopenia (− 2.5 < T ≤ − 1), or osteoporosis (T ≤ − 2.5)

**Fig. 5:**
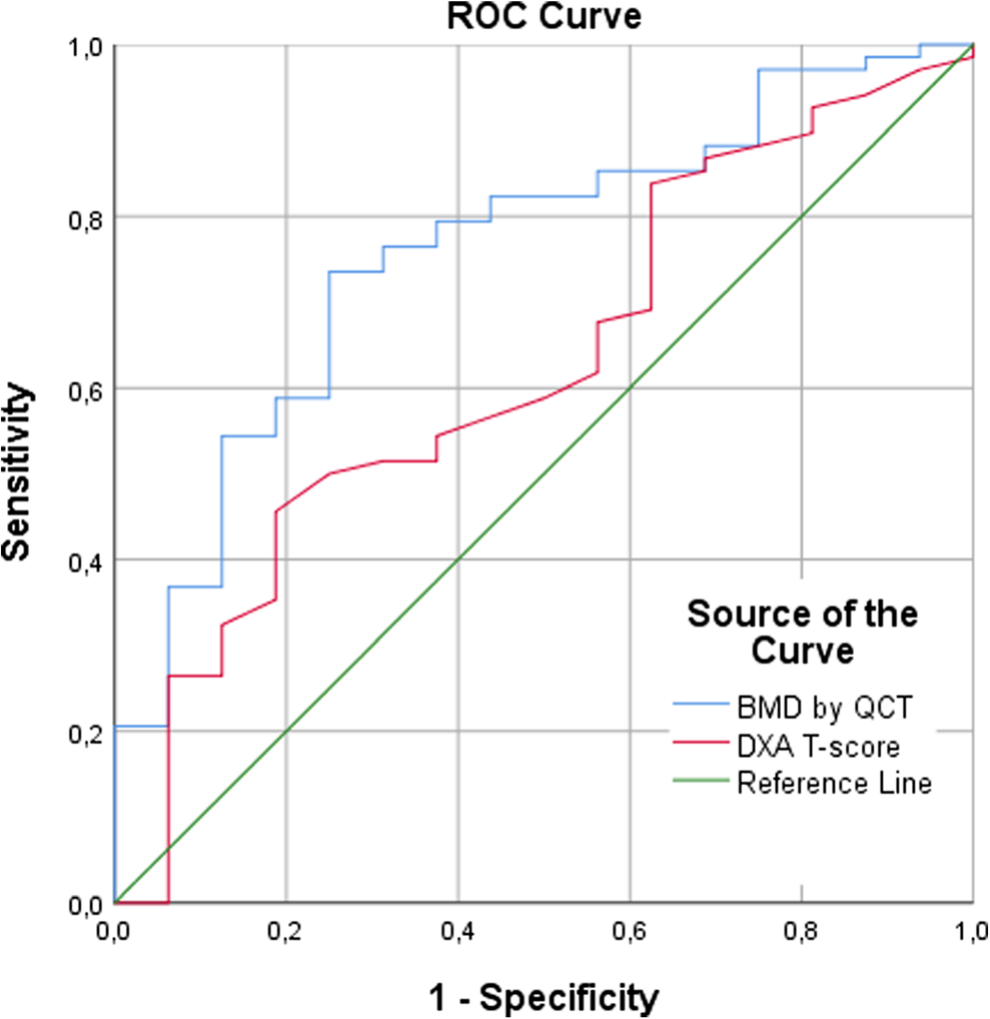
Receiver-operating characteristics curves for predicting incident vertebral fractures by opportunistic QCT (BMD) and DXA (*T*-score)

There was a statistically significant association between the risk of incident vertebral fractures and unadjusted trabecular BMD with a HR of 4.07 per SD decrease in BMD_QCT_ (CI, 1.98–8.38); there was no significant association with unadjusted DXA *T*-score (Table 2, Figs. 3 and 4). The HR for incident vertebral fractures per SD decrease in BMD_QCT_ varied, but remained statistically significant, after adjusting for age (3.60; CI, 1.70–7.64), for age and sex (4.02; CI, 1.83–8.82), and for age, sex, and prevalent fractures (2.54; CI, 1.09–5.90). Only after adjusting for age and sex, a statistically significant increase in HR per SD decrease in DXA *T*-score was observed (1.57; CI, 1.04–2.38). BMD_QCT_ was a significant classifier to predict incident vertebral fractures (AUC = 0.76; CI, 0.64–0.89), DXA *T*-score was not (Table 3 and Fig. 5). BMD_QCT_ values greater than or equal to 79.6 mg/cm^3^ could predict incident vertebral fracture with a specificity of 81% and a sensitivity of 59%.

**Table 2:** Uni- and multivariate (adjusted for age at DXA, sex, and prevalent vertebral fractures) hazard ratios for the risk of incident vertebral fractures

Main variable	Hazard ratio per SD decrease in *T*-score/BMD (CI)			
	Unadjusted	Adjusted for		
		Age	Age and sex	Age, sex and prevFX
*T*-score by DXA	1.36 (0.93–1.99)	1.43 (0.98–2.09)	*1.57 (1.04–2.38)*	1.55 (0.97–2.48)
BMD by QCT	*4.07 (1.98–8.38)*	*3.60 (1.70–7.64)*	*4.02 (1.83–8.82)*	*2.54 (1.09–5.90)*

Statistically significant hazard ratios are in italics. *CI*, 95% confidence interval; *SD*, standard deviation; *prevFX*, maximum grade of prevalent vertebral fractures according to semiquantitative score by Genant

**Table 3:** Classifier performance of BMD_QCT_ and DXA *T*-score for the prediction of incident vertebral fractures in ROC analysis

Classifier	Area under the ROC curve (CI)	BMD cutoff, mg/cm^3^ (sensitivity)			
		Specificity			
		75%	81%	88%	94%
BMD by QCT	*0.76 (0.64–0.89)*	68.2 (74%)	79.6 (59%)	87.0 (54%)	104.8 (37%)
*T*-score by DXA	0.63 (0.48–0.78)	–	–	–	–

Statistically significant area under the ROC curve is in italics

*CI*, 95% confidence interval

Long-term scanner stability was good for all three investigated MDCT scanners. Linear drift was *SL*_*iCT*_ =  − 0.55 HU per year over an observation period of 5.33 years with a *CV*_*iCT*_ = 1.1% for Philips iCT 256, *SL*_*AS*_ =  − 2.29 HU per year over an observation period of 4 years with a *CV*_*AS*_ = 1% for Siemens Somatom Definition AS, and *SL*_*C*64_ =  − 0.81 HU per year over an observation period of 4.09 years with a *CV*_*C*64_ = 0.7% for Siemens Somatom Sensation Cardiac 64 (Table 4).

**Table 4:** HU-to-BMD conversion equations by asynchronous calibration and long-term stability for MDCT scanners used in this study

MDCT scanner	Patients (women)	HU-to-BMD conversion		Long-term stability	
		Calibration phantom	Conversion equations, BMD in mg/cm^3^	Observation period, years	Linear HU change per year (CV)
Philips Brilliance 64	18 (14)	QRM-BDC/3	BMD = 0.778 × HU − 4.693	n/a	n/a
Philips iCT 256	20 (13)	QRM-Abdomen-Phantom	BMD = 0.855 × HU + 1.172	5.33	− 0.55 (1.1%)
Siemens Somatom Definition AS+	4 (4)	QRM-Abdomen-Phantom	BMD = 1.011 × HU − 3.385	n/a	n/a
Siemens Somatom Definition AS	28 (24)	Siemens Osteo*	BMD = 0.985 × HU + 15.516	4.0	− 2.29 (1%)
Siemens Somatom Sensation Cardiac 64	14 (12)	Siemens Osteo*	BMD = 0.971 × HU + 13.249	4.09	− 0.81 (0.7%)

The calibration Phantom marked with an asterisk (*) was situated under the patient in the scanner couch during non-dedicated clinical CT scans (Fig. 2) and retrospectively used for asynchronous calibration. QRM-BDC/3, bone density calibration phantom with 3 rods of defined hydroxyapatite concentration; QRM-abdomen-phantom, anthropomorphic abdomen phantom with 400 × 300 mm obesity extension ring and central insert with 4 rods of defined hydroxyapatite concentrations; *CV*, coefficient of variation of the standard error of the estimate

## Discussion

In this retrospective study, trabecular BMD assessed by opportunistic QCT showed a high association with the risk of incident vertebral fractures in a mixed population of mainly neurosurgical and oncologic patients. In contrast, the association of *T*-scores measures by DXA was non-significant. Only after adjusting for age and sex, *T*-scores were associated with the risk of incident vertebral fracture. Furthermore, more than every second patient (56%) who developed a new osteoporotic vertebral fracture was not diagnosed with osteoporosis according to DXA, whereas the rate of false-negative diagnosis by opportunistic QCT was much lower (19%).

Many cross-sectional studies compared the capability of DXA and conventional QCT to discriminate between patients with and without prevalent spinal fractures [15–18, 31, 32]. A better ability of opportunistic QCT than DXA to classify these patients was suggested as a secondary result in a study, in which 22 out of 37 patients with a prevalent vertebral fracture (59%) had non-osteoporotic DXA *T*-scores [33]. There are further reports when DXA struggled to correctly diagnose approximately every second patient with manifest osteoporosis [34, 35]. The influence of degenerative changes of the spine on the results of DXA is a long known issue [36–38], that can be mostly overcome by QCT [34, 39, 40]. It seems plausible that in our study population the diagnosis of osteoporosis by DXA was less accurate than by QCT, because there was a majority of elderly neurosurgical patients presenting themselves with back pain and most likely showing a degree of spinal degeneration above average.

Longitudinal studies reporting future vertebral fractures are rare, mostly using dedicated quantitative or biomechanical CT in prospective cohorts [41–43], and/or lacking reference DXA scans of the spine [19, 43]. To the best of our knowledge, no study has been conducted comparing non-dedicated (=opportunistic) QCT with DXA regarding the association with the risk of future vertebral fractures. Our results are in accordance with previous findings of similar longitudinal studies. Analyzing the prospective database of men aged 65 years and older (MrOS), a higher association with the risk of new clinical vertebral fractures was found for integral BMD measured by QCT than for areal BMD measured by DXA [41]. The age-adjusted relative hazard for new clinical vertebral fractures increased by 5.7 per SD decrease in integral BMD at the spine, and by 3.2 and 1.8 per SD decrease in areal BMD at the lumbar spine and femoral neck, respectively. Of note, integral volumetric BMD summarizes trabecular and cortical bone in a similar way to areal BMD, but stays independent of bone size and degenerative alterations. A study on the same prospective cohort (MrOS) found an age- and race-adjusted HR of 3.69 for the prediction of clinical fractures of the spine by trabecular BMD by QCT [42]. Areal BMD by DXA at the lumbar spine had also a strong association with the risk of these fractures (HR = 3.57), but DXA at the femoral neck performed similar to our results (HR = 1.95). In ROC analysis, trabecular BMD performed also better (AUC = 0.79) than areal BMD at the spine (AUC = 0.72). In our study, more diagnoses (58%) were based on DXA measurements at the lumbar spine. This could explain why the predictive performance of DXA was substantially worse in both aforementioned statistical measures (age- and sex-adjusted HR = 1.57, AUC = 0.63). As discussed above, this might be due to a selection bias towards neurosurgical patients with above-average spinal degeneration limiting the capabilities of DXA in our population. Recently, the first longitudinal study using opportunistic CT data of multiple scanners without calibration found that L1 vertebral trabecular attenuation blow 90 HU was a significant indicator of decreased fracture-free survival [19].

A BMD_QCT_ cutoff predictive for incident vertebral fractures with 81% specificity (59% sensitivity), found in our data, closely matched the threshold suggested to be equivalent to the WHO diagnostic category for osteoporosis (< 80 mg/cm^3^) [44, 29]. Predefined thresholds can only be used if CT attenuation values are calibrated to a density-reference phantom usually with known hydroxyapatite (HA) concentrations. Otherwise, validated machine-specific cutoff values have to be determined [23]. Opportunistic screening for osteoporosis becomes increasingly popular [14, 45]. In contrast to numerous studies of opportunistic screening, where HU values in thoracic or lumbar vertebra were reported [33–35, 46–50], we used asynchronous calibration to obtain lumbar trabecular BMD. Synchronous calibration with an in-scan phantom as in conventional QCT can be replaced by asynchronous calibration as in the opportunistic setting, if scanner stability is maintained [23].

We performed asynchronous calibration of five different MDCT scanners to allow for opportunistic BMD screening in routine CT exams. In this opportunistic setting, the benefits of conventional QCT can be appreciated without its disadvantages of additional radiation and costs compared to DXA. We developed a protocol for dedicated calibration scans using the same parameters (tube voltage, average tube current, and reconstruction algorithm) and creating a similar geometrical setup of the scanned slice (anthropomorphic abdomen phantom with obesity extension rings and central inserts of known HA concentrations close to the position of the lumbar spine) as in routine scans. In case of the two already decommissioned MDCT scanners, we had to rely on routine scans with an in-plane density-reference phantom, yet we were able to exploit a huge number of scans averaging over a period of more than 4 years. We deemed the eccentric position of the in-plane phantom underneath the patient tolerable, as it was still close to the spine of the patient in supine position. This difference in position probably explains the additional intercept of approximately 15 units in the conversion equations of the two decommissioned Siemens scanners compared to the still operational Siemens scanner (Table 4).

Long-term scanner stability could be shown for three CT devices of two major manufacturers. Good short-term precision and low precision errors of intra-observer [51] and inter-observer reproducibility [26] of asynchronous QCT have been shown before. How to correct for intravenous contrast-enhanced scans in opportunistic QCT is still under debate [14, 45]. We used correction offsets for arterial and portal venous contrast phase from a previous study [28], although these were calculated for a different CT scanner. These minor corrections might be negligible, because they did not seem to affect the accuracy of CT measurements [34] and the overall performance for predicting osteoporosis was similar [52], in previous studies. Moreover, in our data, contrast-enhanced scans were equally distributed between patients with and without incident vertebral fractures (Table 1), thus unlikely to bias the results.

There are limitations to this retrospective observational study. As only a limited cohort of mainly neurosurgical and oncologic patients were analyzed, the results might not be applicable to other patient populations. Additionally, all patients received both MDCT and DXA; thus, osteoporosis was already suggested. This may introduce a selection bias; however, this is exactly the patient population where omitting an additional DXA scan could save time and costs. Loss of follow-up is a possible confounding factor, though independent of the employed densitometric technique.

## Conclusion

Osteoporotic trabecular BMD of lumbar vertebrae assessed by opportunistic QCT was associated with an increased risk of incident vertebral fractures in mainly neurosurgical and oncologic patients aged 50 years and older. In contrast, *T*-scores derived from areal BMD by DXA had a less important effect on the fracture risk than age. Opportunistic screening in CT acquired for other purposes can improve the prediction of future vertebral fractures compared to dedicated DXA exams. The feasibility of pro- and retrospective asynchronous calibration with good long-term stability was shown for multiple MDCT scanners, allowing the scanner independent use of pre-established BMD cutoffs for the diagnosis of osteoporosis.

## Abbreviations

ACR: American College of Radiology
BMD: Bone mineral density
CI: 95% confidence interval
CV: Coefficient of variation of the standard error of the estimate
DXA: Dual energy X-ray absorptiometry
HA: Hydroxyapatite
HR: Hazard ratio
QCT: Quantitative CT
SD: Standard deviation
SL: Slope of linear regression
